# Hepatic and Pancreatobiliary Immune-Related Adverse Events in Patients Receiving Immune Checkpoint Inhibitors: A Multidisciplinary Approach

**DOI:** 10.3390/biomedicines14091964

**Published:** 2026-08-31

**Authors:** Sanja Stojsavljevic Shapeski, Goran Poropat, Iva Skocilic, Petra Puz Britvic, Juraj Prejac, Alojzije Lackovic, Lucija Virovic Jukic, Tajana Filipec Kanizaj, Tajana Pavic, Maja Mijic, Ivica Grgurevic, Tomislav Bokun, Sandra Milic, Milos Lalovac, Anita Skrtic, Petra Dinjar Kujundzic, Ana Ostojic, Frane Pastrovic, Jasna Radić, Dorotea Bozic, Bruno Buric, Marin Golcic, Laura Rados, Sara Matulic, Ema Somen, Neven Ljubicic, Josipa Bilandzic, Maja Kolak, Ana-Marija Bukovica Petrc, Lana Bolf, Sanja Ropac, Vlasta Orlic Karbic, Dragan Trivanovic, Iris Vuković, Ivana Mikolasevic

**Affiliations:** 1 Department of Gastroenterology, Clinical Hospital Center Sestre Milosrdnice, 10000 Zagreb, Croatia; 2 School of Medicine, University of Zagreb, 10000 Zagreb, Croatia; 3 Department of Gastroenterology, Clinical Hospital Center Rijeka, 51000 Rijeka, Croatia; 4 School of Medicine, University of Rijeka, 51000 Rijeka, Croatia; 5 Tumor Clinic, Clinical Hospital Center Rijeka, 51000 Rijeka, Croatia; 6 Department of Gastroenterology, General Hospital Tomislav Bardek, 48000 Koprivnica, Croatia; 7 Department of Oncology, Clinical Hospital Center Zagreb, 10000 Zagreb, Croatia; 8 School of Dental Medicine, University of Zagreb, 10000 Zagreb, Croatia; 9 Department of Gastroenterology, Clinical Hospital Merkur, 10000 Zagreb, Croatia; 10 Department of Gastroenterology, Clinical Hospital Dubrava, 10000 Zagreb, Croatia; 11 Faculty of Pharmacy and Biochemistry, University of Zagreb, 10000 Zagreb, Croatia; 12 Department of Pathology, General Hospital Merkur, 10000 Zagreb, Croatia; 13 Department of Hepatology, Clinical Hospital Center Zagreb, 10000 Zagreb, Croatia; 14 Department of Oncology, Clinical Hospital Center Sestre Milosrdnice, 10000 Zagreb, Croatia; 15 Department of Gastroenterology, Clinical Hospital Center Split, 21000 Split, Croatia; 16 Department of Gastroenterology, General Hospital Sibenik, 22000 Sibenik, Croatia; 17 Department of Anesthesiology, Intensive Care Medicine and Pain Management, Clinical Hospital Center Rijeka, 51000 Rijeka, Croatia; 18 Department of Pulmology, Clinical Hospital Center Rijeka, 51000 Rijeka, Croatia

**Keywords:** immune checkpoint inhibitors, immune-mediated hepatitis, immune-mediated pancreatitis, immune-mediated cholecystitis, immune-mediated cholangitis

## Abstract

Parallel to the increased use of immune checkpoint inhibitors (ICIs), the incidence of immune-related adverse events (irAEs) is also increasing. Almost all organs can be affected by the immune-mediated response, including the liver, pancreas and biliary tract. Immune-Mediated Hepatitis is a common complication of ICI therapy, while cholangitis, cholecystitis and pancreatic involvement are less frequent. Emerging evidence from clinical trials and real-world data series helps us better understand the risk factors, diagnostic challenges and management strategies for these irAEs. With wider use of ICIs, more data are also available on the management of special populations with comorbidities and patients with difficult-to-treat or treatment-resistant adverse events, but larger prospective studies and more robust evidence are required for further recommendations on their management and ICI reintroduction. In clinical practice, a multidisciplinary approach to the management of ICI-mediated complications is advised, with close collaboration among specialties such as oncologists, gastroenterologists or hepatologists, pathologists, radiologists and others. While Immune-Mediated Hepatitis is a common irAE of ICI therapy, pancreato-biliary involvement is often overlooked and underdiagnosed. These irAEs require multimodal management, development of new biomarkers and prediction models to facilitate decision-making and improve patient outcomes.

## 1. Introduction

Immune checkpoint inhibitor (ICI) therapy has made an immense shift in the treatment outcomes of cancer patients [1]. By targeting inhibitory immune pathways such as cytotoxic T-lymphocyte-associated antigen 4 (CTLA-4) and programmed cell death protein 1/programmed death ligand 1 (PD-1/PD-L1), ICIs restore antitumor T-cell activity and produce durable clinical responses [2]. However, nonspecific immune activation may disrupt self-tolerance and lead to a broad spectrum of immune-related adverse events (irAEs). The incidence is increasing due to a broadening of ICI indications, combination and longer duration of therapy. Almost all organs can be affected by the irAEs, including the liver, pancreas and biliary tract [3]. Hepatic involvement is well studied; however, pancreatobiliary complications are still often overlooked, underdiagnosed and inadequately treated. Difficulty arises as they may mimic infection, metastatic disease or other drug-induced injuries, while management frequently requires demanding diagnostic procedures, complex immunosuppressive strategies and interruption of anticancer therapy [4,5,6,7].

Management of these complications has to be a joint effort by a multidisciplinary team consisting of oncologists, gastroenterologists, radiologists, pathologists and other specialties.

This review narratively summarizes the published data, available guidelines on the epidemiology, pathophysiology and immunological mechanisms, predictive biomarkers, clinical presentation, diagnostic evaluation, histological sampling, grading and treatment according to grading, rescue treatments, difficult-to-treat situations and special patient population treatment characteristics for hepatic and pancreatobiliary irAEs of ICIs. A systematic search of PubMed/MEDLINE was performed to identify all studies reporting Immune-Mediated Hepatitis (IMH), Immune-Mediated Pancreatitis (IMP), Immune-Mediated Cholecystitis and Immune-Mediated Cholangitis after ICI therapy exposure. Since the terminology in the literature is heterogeneous, the authors searched manually. They included original articles, review articles, guidelines and case reports, regardless of publication date, covering all topics discussed in this narrative review. The search was last updated on 25 May 2026. We excluded studies if they were editorials, expert opinions, non-English publications, duplicate reports or reports without sufficient patient or study-level data.

## 2. Pathophysiology and Immunological Mechanisms of Hepatic and Pancreatobiliary irAEs

### 2.1. Pathophysiology and Immunological Mechanisms in IMH

Despite growing recognition, the pathogenesis of ICI-induced toxicity remains incompletely understood. Hepatic involvement most commonly presents as IMH, characterized by hepatocellular injury with elevated transaminases and/or bilirubin, and less frequently as Immune-Mediated Cholangitis, which involves bile duct inflammation with a cholestatic biochemical profile. Pancreatic involvement, although less common, presents as IMP, affecting both exocrine and endocrine functions.

The development of IMH is multifactorial and related to the liver’s immunotolerant environment and high antigenic exposure from the portal circulation [8]. Proposed mechanisms include T-cell responses against shared tumor and self-antigens, excessive immune activation with cytokine release, autoantibody formation and possible direct effects of ICIs [9]. ICI therapy promotes the expansion of T helper cells and increases the production of proinflammatory cytokines such as interleukin-2 (IL-2) and tumor necrosis factor α (TNF-α), leading to the activation of cytotoxic T lymphocytes, macrophages, natural killer cells and monocytes. Simultaneously, reduced regulatory T-cell function impairs immune homeostasis and lowers levels of anti-inflammatory cytokines, including interleukin-10 and interleukin-35. Activated CD8+ T cells play a central role by amplifying inflammation and contributing to loss of immune tolerance. Hepatic microenvironmental factors, such as slow sinusoidal blood flow and adhesion molecule expression, facilitate interactions between CD8+ T cells, Kupffer cells and sinusoidal endothelial cells. These interactions promote Fas ligand–Fas signaling and interferon-γ-mediated TNF-α release, increasing hepatocyte susceptibility to apoptosis and resulting in liver injury [10,11]. Mechanistically, IMH differs from autoimmune hepatitis (AIH). AIH is dominated by CD4+ T helper cells with strong B- cell and plasma cell involvement, whereas IMH shows CD8+ T-cell predominance and minimal humoral contribution. Consistent with this, patients with checkpoint inhibitor-induced liver injury typically lack antinuclear antibodies (ANAs) and immunoglobulin G (IgG) elevation [12].

Although considerable progress has been made in understanding the mechanisms of irAEs, it remains unclear why only a minority of patients receiving ICIs develop hepatic, biliary or pancreatic toxicity. In contrast, most tolerate treatment without clinically significant autoimmunity. This suggests that checkpoint blockade alone is not sufficient to induce tissue injury and that additional host- and tumor-related factors are involved. Proposed determinants include host genetic susceptibility, differences in the pre-existing autoreactive T-cell repertoire, tumor antigenicity, the intestinal microbiome and local tissue inflammatory factors, although none has yet been validated as a reliable predictive biomarker [9]. Unlike targeted anticancer therapies, ICIs do not directly recognize tumor cells. Instead, they block inhibitory signaling on T cells, enhancing anti-tumor immunity but also reducing peripheral immune tolerance. Thus, irAEs can be regarded as an unintended consequence of systemic immune activation induced by checkpoint blockade. Because ICIs enhance T-cell function without conferring tumor-antigen specificity, activated T cells may also recognize normal tissues in susceptible individuals [10,11]. Cross-reactive recognition of shared tumor and self-antigens has been proposed as one possible mechanism; however, specific target antigens responsible for immune-mediated liver injury have not yet been identified. Overall, current evidence suggests that IMH results from a combination of loss of peripheral tolerance, aberrant CD8+ T-cell activation and host susceptibility rather than a single pathogenic mechanism. Whilst these mechanisms have been most extensively studied in IMH, similar processes are likely to contribute to Immune-Mediated Cholangitis and IMP, although the relevant organ-specific antigens and pathogenic pathways remain poorly defined [9,10,11,12,13].

### 2.2. Pathophysiology and Immunological Mechanisms in Immune-Mediated Cholangitis

Research on the pathogenesis, imaging features and clinicopathological characteristics of Immune-Mediated Cholangitis remains limited. Current evidence indicates that it is a distinct form of immune-mediated hepatotoxicity, largely driven by T lymphocyte-mediated hypersensitivity. The underlying mechanisms seem to differ slightly between PD-1/PD-L1 inhibitors and CTLA-4 inhibitors. Although both PD-1/PD-L1 and CTLA-4 inhibitors induce immune-related toxicity by enhancing T-cell activity, they act at different stages of the immune response. PD-1/PD-L1 blockade primarily augments peripheral effector T-cell activity in tissues, whereas CTLA-4 inhibition promotes earlier T-cell priming in lymphoid organs. This results in subtly different patterns of immune-mediated injury reported in the literature. Biliary epithelial cells, although capable of metabolizing exogenous compounds, lack key protective systems such as the glutathione redox cycle. They express toll- like receptors (TLRs) and HLA class I and II molecules, but at levels insufficient for efficient antigen presentation. As a result, these cells may trigger T-cell-mediated hypersensitivity responses rather than function as effective antigen-presenting cells [14].

### 2.3. Pathophysiology and Immunological Mechanisms in IMP

The precise mechanisms underlying pancreatic injury caused by ICIs are incompletely understood. Nevertheless, histological analyses of pancreatic biopsies have consistently shown infiltration by CD3+ T lymphocytes, predominantly CD8+ cytotoxic T cells. This likely reflects the disruption of inhibitory signaling pathways by ICIs, resulting in unchecked T-cell activation and subsequent damage to both endocrine and exocrine pancreatic compartments. Additionally, immunohistochemical studies have identified lymphocytic infiltrates expressing cytotoxic markers, including T-cell intracellular antigen 1 and granzyme B [14,15].

Although IMP and IMH often occur simultaneously, there is currently no direct evidence of molecular mimicry between any organ or tumor antigens playing a specific role in the pathogenesis of these diseases [16,17]. Growing evidence indicates that gut microbial antigens may play a role in molecular mimicry and can activate cross-reactive T-cells, triggering a systemic immune response and leading to the development of irAEs [17].

## 3. Immune-Mediated Hepatitis

### 3.1. Incidence, Risk Assessment of IMH and Predictive Biomarkers

Incidence and severity of IMH vary depending on treatment modality and underlying tumor type. Overall, the incidence of IMH in patients receiving mono-immunotherapy ranges from 1 to 7%, while severe (grade ≥ 3) hepatotoxicity occurs in around 1–3% of those patients. In contrast, the incidence in patients receiving combination immunotherapy is up to 13–30%, with grade ≥ 3 incidence up to 6–19% of patients. The higher risk of hepatotoxicity with dual immunotherapy is likely due to synergistic immune activation induced by dual checkpoint blockade [8,18,19].

Across ICI classes, CTLA-4 inhibitors consistently carry a higher toxicity risk than PD-1 and PD-L1 inhibitors, likely because CTLA-4 blockade induces broader immune activation, whereas PD-1/PD-L1 inhibition primarily affects peripheral tolerance [20,21].

Further differences can be identified by analyzing the hepatotoxicity risks of individual drugs. Ipilimumab, an aCTLA-4 inhibitor, demonstrates the highest single-drug hepatotoxic potential, particularly at higher doses, whereas PD-1 and PD-L1 inhibitors are associated with lower rates of hepatotoxicity. Combination regimens, as previously stated, show a bigger risk than any monotherapy, with the highest risk seen with ipilimumab plus nivolumab (Table 1) [22,23,24,25,26,27,28,29,30,31,32,33,34,35,36,37,38,39,40]. This is consistently supported by meta-analyses, which also identify prior ICI exposure as an additional risk factor [21]. The last column in Table 1 represents the cumulative number of reports of three hepatitis-related terms (autoimmune hepatitis, IMH and fulminant hepatitis) identified in the FAERS database. Data are presented descriptively and should not be interpreted as prevalence [41].

Out of all tumor types, melanoma seems to be consistently associated with the highest incidence of IMH, particularly in patients treated with CTLA-4 inhibitors, with grade ≥ 3 hepatitis incidence up to 12%, and combination regimens, whose grade ≥ 3 incidence is up to 20% [22,42].

In hepatocellular carcinoma (HCC), the incidence of IMH is also relatively high in contrast to non-hepatic cancers, with hepatotoxicity up to around 6%, but this interpretation is complicated by the frequent presence of underlying cirrhosis or chronic viral hepatitis, which reduces hepatic reserve and increases susceptibility to immune-mediated injury [37,43].

In non-small cell lung cancer (NSCLC), the incidence of IMH with PD-1/PD-L1 monotherapy is generally low for all-grade (1–5%) and grade ≥ 3 toxicity (1–2%), while combination regimens increase the rate of severe hepatotoxicity (5–10%) [18,26].

In renal cell carcinoma (RCC), IMH occurs in approximately 1–7% of patients receiving monotherapy, with grade ≥ 3 events in around 1–3% [44].

In urothelial carcinoma, treatment with PD-L1 inhibitors such as atezolizumab is associated with all-grade hepatitis in approximately 5–9% of patients and grade ≥ 3 hepatotoxicity in around 1–2%, while those treated with durvalumab showed a much safer profile, with an incidence of immune-mediated hepatotoxicity of 0.3% [32,36].

High tumor mutational burden, commonly observed in melanoma and smoking-related lung cancer, is associated with increased neoantigen load, resulting in stronger immune activation and a higher likelihood of both therapeutic response and immune-related toxicity, including IMH [45]. Similarly, microsatellite instability (MSI) contributes to increased immunogenicity by generating more neoantigens and may predispose to higher rates of immune-related complications [46]. Overall, across tumor types, the incidence of IMH reflects a combination of tumor immunogenicity, underlying liver disease and treatment type, with the highest rates observed in melanoma and combination ICI regimens [47].

Unfortunately, to this day there are no reliable predictive tools or biomarkers that can accurately identify high-risk patients before treatment initiation [47]. Females appear more susceptible to developing IMH, as do younger individuals. The reason for this sex-based difference in incidence remains unclear, while the higher frequency of severe cases in younger patients may be explained by a more robust functional response of both the innate and adaptive immune systems [21,48,49]. Furthermore, the presence of other autoimmune diseases increases susceptibility to IMH, possibly by stimulating pre-activated T-cells and due to the liver’s known immunological sensitivity. Underlying chronic liver disease also raises the risk of IMH, particularly hepatitis B infection and its reactivation, which is considered an independent risk factor. Patients with chronic hepatitis B should therefore be closely monitored and undergo serological testing prior to initiating therapy [50,51].

Additionally, metabolic-associated steatotic liver disease (MASLD) may also increase the risk of developing IMH [52]. In a 2025 retrospective study, MASLD was associated with a 5.6-fold higher risk of PD-1/PD-L1-related drug-induced liver disease (DILI), especially when steatosis coexisted with chronic viral hepatitis or heavy alcohol use [53]. Earlier studies similarly identified MASLD as a risk factor for PD-1 inhibitor-related DILI, possibly reflecting a pre-existing proinflammatory hepatic milieu that lowers the threshold for immune-mediated injury [52,54]. By contrast, alcohol use seems to affect treatment response rather than incidence of IMH, as it was associated with inadequate corticosteroid response in grade 3–4 IMH and required more frequent mycophenolate mofetil (MMF) as second-line immunosuppression [55]. Interestingly, although studies so far are limited and involve small patient cohorts, they do not suggest that pre-existing autoimmune liver diseases are a contraindication to initiating therapy, and no increased incidence of IMH has been observed. However, further research on larger patient populations is needed [56].

Among the most frequently investigated predictive biomarkers are autoantibodies, namely antinuclear antibodies, rheumatoid factors and antithyroid antibodies, that have been linked to an increased incidence and severity of organ-specific irAEs following ICI therapy. However, their association with IMH is inconsistent and limited, making them for now unreliable as standalone predictors [57,58]. Proinflammatory cytokines have also been extensively studied, and elevated levels have been observed in more severe cases of IMH. Nevertheless, these findings have not reached statistical significance, and the timing of sample collection appears critical [59]. This underscores the need for longitudinal monitoring and more detailed studies to clarify their role in predicting IMH.

Overall, currently investigated predictive factors and biomarkers can be broadly categorized according to the strength and maturity of the available evidence. Some clinical and immunological factors have demonstrated relatively consistent associations with IMH and may therefore be considered better-supported candidate markers. However, even these markers have not yet undergone sufficient prospective validation to support their use as established clinical predictors. In contrast, emerging or exploratory biomarkers, including specific immune-cell populations, cytokine signatures and genetic variants, have shown promising associations in initial studies but require replication in larger, independent cohorts before their predictive value can be established [60]. The current literature therefore supports a distinction between relatively better-supported clinical risk factors and emerging biomarker candidates, rather than the designation of any biomarker as clinically established. This distinction is particularly relevant because recent reviews emphasize that, despite numerous promising candidates, no biomarker has yet demonstrated sufficient reliability for routine clinical use [48].

The most specific biomarker identified so far is a circulating activated CD8+ effector memory T-cell subset, which correlates with liver injury severity and differentiates IMH from other immune-mediated liver diseases [61,62]. Additional signals, including monocyte activation markers such as soluble cluster of differentiation 163—sCD163, as well as proinflammatory cytokine and chemokine signatures, may further support diagnosis and disease monitoring [61,63,64].

Among these, circulating activated CD8+ effector memory T-cell subsets represent one of the most promising emerging immunological biomarkers identified to date, given their association with liver injury severity and their potential to distinguish IMH from other immune-mediated liver diseases [62]. In particular, circulating exhausted CD8+ effector memory cells expressing CD38, HLA-DR and CXCR3 were able to differentiate checkpoint inhibitor-induced liver injury from other acute immune-mediated liver injuries. Nevertheless, these findings should currently be regarded as promising rather than established, as the study itself highlights the absence of a specific biomarker currently available for routine clinical differentiation of these conditions [62]. Other immunological markers, including monocyte activation markers and cytokine or chemokine signatures, should currently be regarded as exploratory candidates because their clinical utility, reproducibility and prospective predictive value have not yet been sufficiently validated [48].

Genetic factors, particularly certain HLA haplotypes and single-nucleotide polymorphisms (SNPs), may play a key role in modulating immune responses. However, their predictive value also requires validation in studies involving larger patient cohorts [65,66,67,68].

Similarly, genetic associations should currently be considered exploratory rather than as established predictive biomarkers. Although specific HLA haplotypes and SNPs have been associated with susceptibility to IMH, the available evidence is derived largely from relatively small cohorts and requires confirmation in larger, independent populations. For example, a study of 57 high-causality cases of IMH identified associations with EDIL3, SEMA5A, GABRP, SMAD3 and SLCO1B1 variants, whereas HLA alleles traditionally associated with autoimmune hepatitis were not overrepresented [69]. These findings support a potential genetic contribution to susceptibility but remain insufficient to establish genetic variants as clinically useful predictive biomarkers.

For clarity, the investigated predictive factors and biomarkers are categorized in Table 2 according to the current strength of evidence as better-supported, emerging/exploratory or inconclusive. Importantly, the designation “better-supported” does not imply that these factors are clinically established biomarkers or sufficiently validated for routine risk stratification. Rather, it indicates that the available evidence is comparatively more consistent than for emerging or exploratory candidates. This approach is consistent with the recent literature emphasizing that, although several clinical risk factors and biomarker candidates have been identified, prospective validation and large-scale studies are still required before they can be incorporated into routine clinical prediction models [48,60].

### 3.2. Clinical Presentation

The clinical presentation of IMH varies widely, ranging from asymptomatic cases, presented with mild elevations of aminotransferases to severe acute liver failure [10,70]. Furthermore, the clinical presentation may differ depending on the type of ICI used [10]. The severity of IMH is usually graded according to the common terminology for clinical adverse events (CTCAEs) criteria [71]. However, the DILI network criteria may also be considered, as they include clinical symptoms and indices of organ failure, particularly the international normalized ratio (INR) [72]. Additional research should be conducted to determine the explicit criterion for IMH [47]. Severe toxicity occurs in less than 2% of cases but with increased incidence in combination therapy [73]. The most frequent presentation of IMH is asymptomatic hepatitis, characterized by elevated serum aminotransferases, aspartate aminotransferase (AST) and alanine aminotransferase (ALT), detected during routine laboratory testing performed prior to each treatment cycle. Liver injury tends to develop within 6 to 14 weeks after initiation of ICI therapy, often corresponding to the first of three treatment cycles, although earlier or later presentations may occur [74]. When symptoms are present, they are usually nonspecific and may include fatigue, malaise, anorexia (17.1%), fever, nausea and vomiting (14%) [75]. Some patients may also experience abdominal discomfort (11.6%), particularly pain in the right upper quadrant. A minority of patients may present with myalgia and arthralgia [9]. Although acute liver failure is rare, especially as an initial presentation, liver injury may progress in some cases, leading to signs of cholestasis or hepatic dysfunction such as jaundice, dark urine, pruritus or pale stools. In more advanced cases, patients may develop coagulopathy or hepatic encephalopathy, indicating significant hepatic impairment [76]. Although acute liver failure is exceptionally rare, with an estimated incidence of 0.07–0.2% among patients treated with ICIs, it carries a high mortality once established. Nevertheless, the true incidence of ICI-related acute liver failure and its mortality remain poorly defined owing to selective reporting and frequent discrepancies in the diagnostic criteria and classification of acute liver failure [77]. Although an exact mortality rate for ICI-associated acute liver failure cannot be reliably established, the best available estimate comes from the largest available pharmacovigilance analysis, where 449 of 654 reported cases of ICI-associated hepatic failure (68.65%) were fatal. However, this represents the fatality proportion among reported cases and should not be interpreted as a population-based mortality rate due to the inherent limitations of FAERS data [78]. Due to the potential for delayed onset of IMH, with cases reported up to 12 months after the last dose of immune checkpoint inhibitor therapy, continued clinical awareness and consideration of liver function monitoring may be warranted after treatment discontinuation. Although the mechanisms underlying delayed IMH remain incompletely understood, they may be related to the prolonged immunological effects of checkpoint inhibition, which can persist beyond drug discontinuation. Therefore, IMH should remain part of the differential diagnosis in patients presenting with liver injury even months after cessation of ICI therapy [79].

### 3.3. Diagnostic Workup, Differential Diagnosis and Role and Timing of Liver Biopsy with Histological Features

All patients receiving ICIs who develop symptoms and/or elevated liver enzymes should undergo a structured diagnostic evaluation to exclude alternative causes of liver injury (Table 3). The differential diagnosis of IMH includes hepatic metastases or infiltrative malignant disease, biliary tract obstruction, thromboembolic or other vascular disorders, ischemic or congestive liver injury, opportunistic infections, viral hepatitis, autoimmune liver disease and hepatotoxicity from concomitant drugs or other xenobiotics. Diagnostic workup should include careful assessment of prior liver disease, current and previous medications, alcohol intake and dietary supplements, together with serological testing for viral and autoimmune liver disorders and imaging of the hepatobiliary system and hepatic vasculature [5]. Serial monitoring of liver biochemistry remains standard of care at treatment initiation and before each treatment cycle. Abdominal imaging is performed primarily to rule out competing etiologies. Findings in IMH are usually nonspecific and may include mild hepatomegaly, decreased liver attenuation on computed tomography, hypoechogenic liver parenchyma on ultrasound or periportal lymphadenopathy [80]. In cholestatic presentations, ultrasound, contrast-enhanced computed tomography (CT), magnetic resonance imaging (MRI), magnetic resonance cholangiopancreatography (MRCP) or endoscopic ultrasound (EUS) may be used to assess biliary obstruction, whereas endoscopic retrograde cholangiopancreatography (ERCP) is reserved for selected interventional indications. Liver biopsy should be considered in patients with an uncertain diagnosis, in those who fail to improve after 3–5 days of corticosteroid therapy or in cases of grade 3–4 IMH. Liver biopsy can be performed percutaneously or under guidance of EUS. As a procedure, EUS is multifunctional and can be a noninvasive and invasive diagnostic method. The biopsy specimens obtained with EUS have proven to be noninferior to conventional liver sampling and in some studies achieved an even better diagnostic yield when assessed by a designated liver pathologist [81,82].

Liver biopsy in IMH most often shows an acute hepatitis-like, predominantly lobular pattern with spotty or confluent necrosis, acidophil bodies and frequent centrilobular (zone 3) accentuation, sometimes with lobular disarray and parenchymal collapse [10,47,83]. Portal inflammation and interface activity are variable and often less prominent than in AIH, while perivenular infiltrates, centrilobular necrosis and occasional eosinophils or histiocytes may be present [10,47,83]. Immunophenotyping typically demonstrates a predominance of activated CD3+/CD8+ T lymphocytes with fewer CD4+ T cells, CD20+ B cells and plasma cells than classical AIH, with minimal plasmacytosis [10,47,84]. Granulomatous hepatitis, including microgranulomas and fibrin-ring granulomas with fibrin deposits and central vein endothelitis, has been reported particularly with CTLA-4 inhibitor-containing regimens [10,47,83]. Less commonly, a cholestatic or mixed phenotype occurs, with portal mononuclear infiltrates centered on bile ducts and ductular proliferation, underscoring overlap with other drug-induced liver injury patterns and the lack of a pathognomonic lesion [10,83,85].

We recommend that when liver biopsy in this setting is required, the analysis should be conducted in a center with designated liver pathologists regarding the complexity of differential diagnosis.

### 3.4. IMH Severity and Grading

Severity is graded according to the National Cancer Institute CTCAE, which classifies hepatotoxicity into five grades based on the degree of liver test abnormality and the presence of liver failure or portal hypertension-related complications (Table 4) [71]. However, although CTCAE-based grading is shared with other forms of hepatotoxicity, IMH differs from conventional hepatitis and classical DILI because grading reflects biochemical severity rather than the specific immune-mediated mechanism, clinicopathological context and treatment implications of this entity. In IMH, interpretation of grade must therefore be integrated with exposure to ICIs, exclusion of competing etiologies and consideration of immunosuppressive therapy.

### 3.5. Therapy of IMH

#### 3.5.1. Treatment According to Severity

IMH treatment is based on stepwise immunosuppression, with corticosteroids as first-line therapy and escalation to second-line immunosuppressants in steroid-refractory cases (Table 5) [18]. Up to half of patients with grade 3–4 hepatitis may show inadequate corticosteroid response, with male sex and alcohol use associated with poorer outcomes [21]. In grade 1 disease, ICIs may be continued with close monitoring (liver function tests (LFTs) 1–2×/week) and corticosteroids are not required. If transaminases show a concerning upward trend, temporary ICI hold should be considered [18].

Grade 2 hepatitis necessitates temporary ICI interruption. ICI and liver function tests should be monitored every 3–5 days with periodic INR assessment. Corticosteroids (prednisone 0.5–1 mg/kg/day) should be considered if symptomatic or if no improvement occurs within 1–2 weeks. ICI may be resumed once hepatitis resolves to grade 1 and corticosteroids are tapered to ≤10 mg prednisone equivalent daily [18].

In grade 3, ICI should be held and corticosteroids initiated at prednisone/methylprednisolone 0.5–1 mg/kg/day (maximum 60 mg/day) [18]. Importantly, a recent study demonstrated that initial treatment with prednisone/methylprednisolone 1 mg/kg/day provides similar hepatitis outcomes compared to higher-dose regimens (≥1.5 mg/kg/day), with significantly reduced steroid-related complications including infections and hyperglycemia requiring treatment [86]. If no improvement occurs after 1–2 days, second-line immunosuppression should be added.

In grade 4 hepatitis, ICI should be permanently discontinued and patients should be hospitalized [18]. Corticosteroids should be initiated at methylprednisolone 1–2 mg/kg/day IV, with consideration of early concomitant MMF at initiation. Cholestatic patterns require exclusion of biliary obstruction and early addition of ursodeoxycholic acid (UDCA), as they are associated with poorer outcomes [18].

#### 3.5.2. Steroid-Dependent and Steroid-Refractory IMH

The lack of biochemical improvement in serum transaminase levels within 2–3 days of treatment defines steroid-refractory IMH [4]. Steroid-dependent IMH is defined as the occurrence of a biochemical flare during the tapering of corticosteroid therapy and it requires dose escalation (methylprednisolone 2 mg/kg/day) until initial regression of liver enzymes is achieved, followed by a gradual dose reduction over a period of 4 to 6 weeks [4,5,10].

In both steroid-dependent and steroid-refractory IMH, the absence of a biochemical response to maximum-dose methylprednisolone (2 mg/kg/day) within the first 48–72 h indicates the need for alternative immunosuppressive therapy [4,5,18].

MMF is the preferred second-line agent due to its favorable safety profile and rapid therapeutic onset [12,87]. It is typically administered at a dose of 500–1000 mg twice a day, concurrently with corticosteroids [4,5,6,88,89,90]. If there is a regression of liver enzymes after 7–10 days, corticosteroids are gradually tapered over 4–6 weeks [91]. Currently, there are no established recommendations for MMF dose reduction. Tapering is highly individualized, lasting from a few weeks to over six months and the choice between a rapid or prolonged approach depends on the severity of liver injury, the initial response and the risk of relapse [92].

Tacrolimus is recommended as a third-line option, though it can be used in the second line for patients with concomitant leukopenia and diarrhea [6]. It is administered at a dose of 1.5–4 mg twice a day, aiming for a serum concentration of 5–8 ng/mL. If an improvement in liver enzymes is achieved, tacrolimus can be tapered by 0.5 mg weekly, but only after the successful discontinuation of MMF and corticosteroids [93,94].

**Figure 1:**
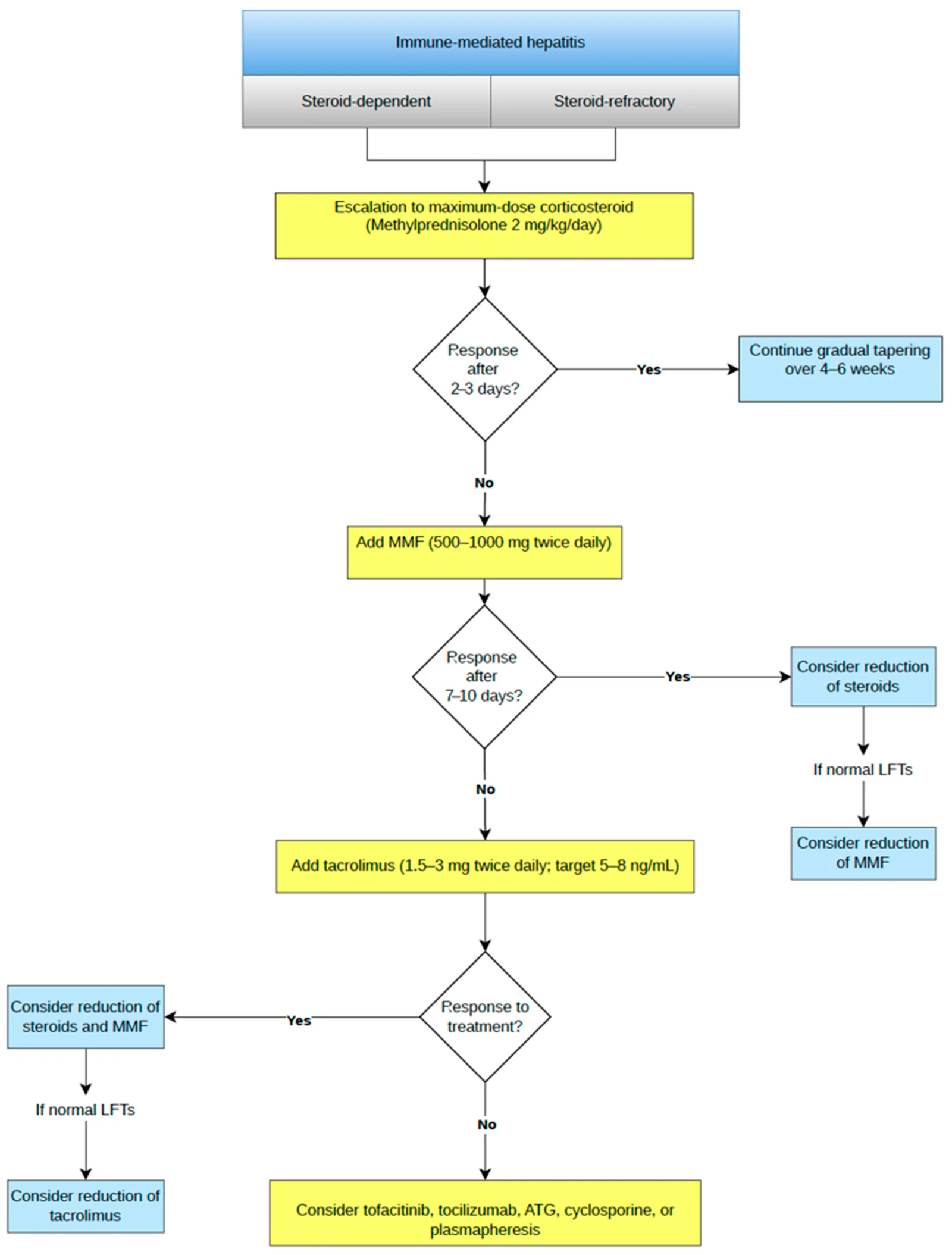
Treatment flowchart for IMH in cases of steroid-dependent and steroid-refractory disease (MMF—mycophenolate mofetil; ATG—anti-thymocyte globulin).

Alternative immunosuppressive therapeutic options include tocilizumab, cyclosporine, anti-thymocyte globulin (ATG), tofacitinib and plasmapheresis [95,96,97,98], whereas infliximab (IFX) and azathioprine are generally avoided due to safety concerns and slow efficacy [4,5,7,9,18]. As already stated, if laboratory results in a refractory period show a dominant cholestatic pattern, UDCA can be added to the therapy [99]. In Figure 1 is a proposed treatment diagram for steroid-dependent and steroid-refractory cases (Figure 1).

#### 3.5.3. De-Escalation of Therapy

In the grade 2 IMH, corticosteroid tapering should be started after achieving grade 1, with a gradual dose reduction over 2–4 weeks. Regarding grades 3 and 4, after reducing inflammation to second grade, conversion to an oral equivalent of corticosteroid should be conducted, with further tapering until discontinuation over 4–6 weeks [4,5]. The optimal strategy for corticosteroid dose reduction is unknown. Most recommendations are based on expert opinion with the lack of evidence from prospective medical studies. On one hand, a faster reduction in the corticosteroid regimen is desirable in order to avoid side effects, although, on the other hand, swift discontinuation may trigger a relapse and even development of adrenal insufficiency. Furthermore, the question arises of whether corticosteroid therapy and the duration of its use affect the efficacy of immunotherapy itself. Studies to date are scarce, but it appears that the use of corticosteroid therapy, indicated for the treatment of immunotherapy side effects, has no impact on its efficacy, progression-free survival and overall survival in this group of patients [100,101]. The guidelines of European and American Societies of Oncology suggest somewhat different corticosteroid dose de-escalation strategies; over 2 weeks in the case of initial grade 2 according to European guidelines, versus 4 weeks according to the American guidelines. In grades 3 and 4, European guidelines suggest beginning of dose reduction after achieving grade 2 over a period of 4 weeks, while American guidelines recommend a slightly longer de-escalation period of 6 weeks, after achieving grade 1 [4,5]. When using multiple immunosuppressive agents, they should be tapered one at a time rather than simultaneously.

#### 3.5.4. Reintroduction of Immune Therapy and Recurrence of IMH

Patients receiving ICIs are typically those with advanced malignancies and limited therapeutic options, which raises the key question of whether ICI therapy should be reintroduced after the development of IMH. According to major oncology society guidelines, ICI treatment should be temporarily withheld in cases of grade 2 hepatitis until improvement to grade 1 or complete normalization of liver function tests, and permanently discontinued in cases of grade 3 or 4 hepatitis [4,102]. With the increasing use of ICIs, a growing number of case reports and studies report successful rechallenge even after grade 3 or 4 hepatitis. IMH develops in approximately 22% to 35% of cases following ICI rechallenge, and the majority of these are non-severe [88,98,103,104].

There also seem to be differences by ICI class, in that rechallenge with anti-PD-1/PD-L1 monotherapy appears to carry a significantly lower risk of hepatitis recurrence than anti-CTLA-4 therapy (e.g., ipilimumab) [105,106].

All studies emphasize an individualized approach, with potential benefits outweighing the risks, and close patient monitoring [98,107]. A modification of the therapeutic regimen is also recommended when reintroducing treatment by switching from one therapeutic agent to another [79]. The question of prophylactic immunosuppressive therapy prior to reintroduction of ICI naturally arises in clinical practice; however, there is currently no clear evidence supporting its routine use. Although some studies suggest that budesonide may be safe when administered alongside ICI rechallenge, further research is needed before firm recommendations can be made [108].

### 3.6. Special Clinical Situations

Patients suffering from viral infections such as hepatitis B (HBV), hepatitis C (HCV) or human immunodeficiency virus (HIV), as well as those with end-stage liver disease of any etiology and transplant recipients, are most often a priori excluded from clinical trials, resulting in a lack of sufficient data and clear guidelines for their management [109]. In these liver diseases, the hepatocellular reserve is reduced, and hepatocytes are more susceptible to immunological stress, thereby increasing the risk of developing IMH.

#### 3.6.1. Patients with HBV

Before initiating ICI therapy, it is necessary to determine HBV serology, given that reactivation of chronic HBV infection is possible and is considered an independent risk factor for the development of complications such as IMH. However, when the reactivation occurs, it is hard to determine the cause of the liver injury and in that case viral hepatitis infection or reactivation is the main exclusion criterion for IMH. It is also possible that reactivation may influence the efficacy of ICI therapy as well as overall survival [50].

In individuals with negative HBV markers, vaccination against hepatitis B is recommended, whereas in those with positive HbsAg, treatment with entecavir or tenofovir should be initiated and continued for at least 12 months after completion of ICI therapy [110,111]. The incidence for the HBV reactivation varies among different studies; in a published systematic review by Asian authors, the risk of HBV reactivation was reported to be approximately 1.3% [112]. In patients who are HbsAg-negative and anti-HBc-positive, HBV DNA levels should be determined and treatment planned accordingly [111]. The approach to these patients should be individualized, with closer monitoring of laboratory parameters and HBV DNA levels.

#### 3.6.2. Patients with HCV

Most published data found no association between ICI therapy and HCV reactivation [113,114,115,116,117,118,119,120]. Notably, it was shown that ICIs alone, even without direct-acting antivirals (DAAs), can inhibit HCV replication and lead to virologic cure [121]. HCV reactivation has occurred in one study in 4% (2/52) of patients, and during concurrent use of immunosuppressants for ICI-related toxic effects, making it difficult to attribute the reactivation solely to ICI therapy [122]. There are no significant differences in rates and severity of adverse events between patients with chronic HCV infection and those without who are receiving ICI treatment. Objective responses and disease control in HCV-infected patients were similar to non-infected patients. Currently available data suggest that ICI therapy may be a therapeutic option for patients with cancer and chronic hepatitis C, with an acceptable safety profile and antitumor activity [109]. However, careful monitoring for hepatitis flare and reactivation is recommended, especially in cases of concurrent immunosuppressive therapy use. Current evidence suggests that DAAs are safe and can eradicate HCV when administered before, during or after ICIs. A personalized approach to HCV management is essential for cancer patients on ICI therapy [109].

#### 3.6.3. Patients with HIV

PLWH (people living with HIV) and malignancies are another population underrepresented in ICI clinical trials, despite their increased risk of developing various cancers [123]. According to retrospective studies, the presence of HIV infection is not associated with a difference in efficacy of ICI therapy nor with an increased incidence of irAEs compared to the general non-HIV-infected population [124,125]. Furthermore, ICI therapy in PLWH could promote HIV latency reversion, but further research is necessary [126,127].

#### 3.6.4. Patients with Cirrhosis

ICIs may be used in carefully selected patients with cirrhosis, particularly those with HCC, but treatment requires close attention to hepatic reserve and baseline liver dysfunction considering the higher risk of hepatotoxicity in patients with cirrhosis, autoimmune liver disease or liver metastases [128,129,130]. In practice, the best candidates are patients with preserved liver function, most commonly Child-Pugh A score, good performance status and no extrahepatic spread when ICIs are considered for downstaging or bridging to liver transplantation (LT) [131,132]. Current data suggest that ICIs before LT can achieve meaningful tumor control and may enable transplantation in selected patients initially beyond Milan criteria; however, the main safety concern is acute graft rejection after transplantation [132,133]. For this reason, a washout period of at least 6–8 weeks, and preferably about 12 weeks or at least 90 days, is recommended before LT [132,134]. After LT, ICIs are not routinely recommended for recurrent HCC because acute rejection occurs in approximately 30–50% of cases and may lead to graft loss or death [135,136]. Post-transplant ICI therapy should therefore be reserved for highly selected patients after exhaustion of other oncologic options and under strict multidisciplinary supervision [136,137].

## 4. Pancreatobiliary Complications

### 4.1. Incidence, Risk Assessment and Potential Biomarkers of Pancreatobiliary Complications

Immune-mediated pancreatobiliary toxicities are uncommon but clinically relevant irAEs of ICIs. The incidence of pancreatic injury varies depending on definitions and study design. Pancreatic adverse effects were observed in 1.1–3.7% of patients, ranging from asymptomatic enzyme elevations to pancreatitis and autoimmune diabetes [138]. In a systematic review, overall incidence of IMP was reported to be 0.93%, while asymptomatic elevations of amylase (2.57%) and lipase (2.78%) were more frequent [139]. Furthermore, reported pooled incidence of pancreatic injury was 2.22%, with higher rates in combination therapy (3.76%) compared to monotherapy (2.25%), and grade ≥ 3 events occurring in approximately 2% of patients [140]. Real-world data suggest slightly higher incidences of IMP, observed in 5.7% of patients, although symptomatic and severe cases remained rare (up to 0.63%) [141].

Therapy type appears to be a potentially important risk factor. Combination ICI therapy in certain cases demonstrated higher toxicity rates, probably due to enhanced T-cell activation, whereas monotherapy seems to be associated with a lower risk. Among monotherapy regimens, PD-L1 inhibitors as a class may be associated with higher rates of pancreatic injury. The most data in this context are available on pembrolizumab, which possibly indicate a higher risk of pancreatic toxicity, but this is still only an observation [140,142,143]. Prolonged exposure (≥10 cycles) further increases the risk of pancreatic toxicity [141].

Risk may also vary by cancer type, although data remain limited and sometimes inconsistent. Some studies suggest a higher incidence of pancreatitis in melanoma patients treated with CTLA-4-containing regimens than in patients with other malignancies. Pancreatic and lung cancers have also been associated with an increased risk of severe pancreatic adverse events, likely reflecting both disease- and treatment-related factors [142,143].

Biliary complications are less frequent but increasingly recognized. Immune-Mediated Cholangitis occurs in approximately 0.05–0.7% of patients, although the true incidence, particularly of small-duct disease, is likely underestimated [144]. Up to 40% of ICI-mediated biliary complication cases are associated with concurrent irAEs, most commonly colitis [145]. These events are most often reported with PD-1 inhibitors, although PD-L1 and CTLA-4 inhibitors are also implicated [146,147,148]. Immune-Mediated Cholecystitis is rare (approximately 0.6%), and vanishing bile duct syndrome is an exceptionally rare manifestation, described only in a few case reports [149,150,151].

Treatment-related factors, particularly combination therapy and prolonged exposure, remain the most consistent predictors of ICI pancreatobiliary complications [140,141,143].

Currently, no validated predictive biomarkers for pancreatobiliary irAEs have been established. However, emerging data suggest that elevated baseline eosinophil count and C-reactive protein, as well as PD-1 inhibitor therapy, may be associated with an increased risk of Immune-Mediated Cholangitis [152]; however, these should still be regarded as exploratory due to a lack of solid scientific evidence. A prior history of pancreatitis may also predispose patients to pancreatic toxicity [138].

### 4.2. Clinical Presentation

Pancreatobiliary complications may occur at any stage of ICI treatment but most commonly develop within the first few months, typically between 4 and 12 weeks after treatment initiation [153,154].

The clinical presentation is usually mild and resembles conventional acute pancreatitis, with symptoms such as epigastric abdominal pain radiating to the back, nausea, vomiting, fever and diarrhea [155,156]. Epigastric pain is the most frequent symptom, occurring in approximately 39% of cases, while severe presentations are rare [155].

A substantial proportion of patients remain asymptomatic despite biochemical or radiological evidence of disease. Between 61% and 86% of patients with elevated amylase and lipase levels have no symptoms, and asymptomatic enzyme elevation occurs in 2.7–10.6% of patients receiving ICIs [15,155,156]. Imaging studies may also show features of pancreatitis in the absence of clinical manifestations [156].

Pancreatic involvement may present solely with metabolic disturbances. Hyperglycemia and symptoms of diabetes may be the only clinical manifestation, while exocrine pancreatic insufficiency can develop independently of radiological changes or enzyme elevation [15,157].

Chronic manifestations are less common but clinically relevant, including persistent abdominal pain and progressive pancreatic damage leading to exocrine and endocrine insufficiency, often without fulfilling classical criteria for chronic pancreatitis [15,158,159].

Immune-Mediated Cholangitis typically presents with right upper quadrant pain, fever, jaundice and systemic symptoms such as fatigue, nausea and pruritus. Immune-mediated cholecystitis resembles the conventional form and generally develops later, with a median onset of approximately 6 months after ICI initiation [144,146,147,148,149,150].

Asymptomatic presentations are less frequently reported in immune-mediated cholangitis, although mild liver enzyme elevations without symptoms may reflect subclinical biliary involvement detectable only by imaging [156].

### 4.3. Diagnostic Workup and Differential Diagnosis, Role and Timing of Pancreatic Biopsy with Histological Features

The diagnosis of pancreatobiliary complications of ICI therapy relies on a multimodal approach integrating imaging, clinical presentation and laboratory data [156]. Asymptomatic increases in pancreatic enzymes are a known occurrence during ICI therapy and are not always diagnostic of pancreatitis. Friedman et al. observed that among patients with grade 3 or higher enzyme elevations, IMP was confirmed in only 20% of those with hyperamylasemia and 6.7% of those with hyperlipasemia [160].

Based on revised Atlanta classification from 2012, IMP is diagnosed when at least two out of the three diagnostic criteria (clinical, biochemical, imaging) for acute pancreatitis are met [160]. The criteria are shown in Table 6 with the most common imaging findings [4,155,158,161,162,163]. Chronic pancreatitis and autoimmune pancreatitis (AIP) represent the most common differential diagnoses to be considered when IMP is suspected, owing to their clinical and imaging aspects, and thus demand a structured and advanced diagnostic approach, as shown in Table 6 [157,160,164,165,166].

Imaging examination is crucial for the diagnosis and differential diagnosis of Immune-Mediated Cholangitis. Given this mimicry, a broad differential diagnosis is essential and it must be distinguished from various causes of intrahepatic and extrahepatic cholestasis, ranging from autoimmune conditions (primary sclerosing cholangitis (PSC), primary biliary cholangitis (PBC), IgG4-related sclerosing cholangitis (IgG4-SC)) to malignant obstructions, infections and drug-induced lesions [145]. Patients with Immune-Mediated Cholangitis may present with changes confined to the large bile ducts (large-duct type) or with a mixed pattern involving both large and small bile ducts. The main imaging features include segmental or diffuse dilatation, stenosis and irregular thickening of the bile duct walls, which may occur at different anatomical levels of the biliary tree: isolated intrahepatic and extrahepatic bile ducts, or both. Imaging findings frequently overlap with IgG4-SC and PSC. Features such as distal duct beading and extrahepatic wall hypertrophy suggest an IgG4-SC-like phenotype, whereas intrahepatic multifocal stenoses suggest a PSC-like phenotype. This allows for a two-tier radiographic classification based on which established disease pattern the ICI induced injury most closely resembles [157]. The use of PET-CT in this setting is very rare and limited to a few case reports showing increased fluorodeoxyglucose uptake in the gallbladder and bile ducts [145]. Follow-up imaging during the course of the disease is recommended 1.5–3 months after initiation of immunosuppressive therapy for Immune-Mediated Cholangitis, and between 1 and 7 months (on average 3 months) for pancreatitis, until resolution of inflammation is confirmed [145,162].

Liver biopsy is not a routine method in the diagnostic work-up of cholangitis caused by ICI therapy. Its main role is to confirm the diagnosis of small-duct cholangitis, exclude other causes and guide therapy when non-invasive methods are insufficient. The primary indication for biopsy is clarification of the cause of liver injury in patients with atypical findings or an atypical clinical course. Liver biopsy is the gold standard for the diagnosis of ICI -related small-duct cholangitis. However, due to its invasiveness and potential complications, routine biopsy is not recommended in mild and typical clinical cases confirmed [145]. The American Society of Clinical Oncology guidelines recommend considering liver biopsy in all grade 3 and grade 4 liver injuries [5].

Pancreatic biopsy in the context of IMP is performed exclusively under EUS guidance. The primary indication is to confirm or exclude malignancy in the presence of a newly detected focal mass or a suspicious lesion on CT or MRI. EUS fine-needle biopsy (EUS-FNB) is the gold standard because it enables better evaluation of tissue architecture. ESGE recommends end-cutting FNB needles over reverse bevel FNB or fine-needle aspiration (FNA) needles for tissue sampling of solid pancreatic lesions [167]. In cases where the expected response to therapy is lacking, particularly in severe pancreatitis, pancreatic biopsy may be considered in order to exclude other causes of disease. In the absence of high-quality prospective studies, this decision remains at the clinician’s discretion. When clinical and radiological findings suggest possible AIP unmasked by ICI therapy, FNB is crucial. Confirmation of AIP requires classical histological features which can only be identified in a tissue specimen. The overall diagnostic accuracy rate with end-cutting EUS-FNB needles in patients with AIP is 80% [168].

Published data on the histopathological diagnosis of IMP are very limited and restricted mainly to case reports. Histopathological specimens obtained during surgery or by EUS-FNB show marked infiltration of pancreatic islets by CD3+, CD4+ and CD8+ T lymphocytes, without evidence of malignancy. A high CD8+/CD4+ T-lymphocyte ratio is also typical, a finding consistent with irAE [142,164].

The characteristics of the histopathological diagnosis of Immune-Mediated Cholangitis depend on the type of specimen. When liver biopsy is performed, findings indicate inflammatory cholangiopathy at the level of the portal tracts, most commonly bile duct injury, in approximately half of cases a ductular reaction and lobular injury, and less frequently cholestasis and bile duct loss. Biopsy of extrahepatic bile ducts is rarely performed, but histopathology may reveal inflammatory infiltration of the overlying epithelium and diffuse fibrosis in approximately one-third of samples. A characteristic feature of Immune-Mediated Cholangitis is also marked CD8+ T-lymphocyte infiltration with a high CD8+/CD4+ T-lymphocyte ratio [145,169].

### 4.4. Classification

In clinical practice, severity is most commonly assessed using the CTCAE, which guides decisions on the continuation, modification or discontinuation of ICI therapy [71,156].

Grading is based on an integrated evaluation of clinical presentation, laboratory findings, imaging and treatment response, but primarily reflects symptom severity and need for intervention. Grade 2 includes asymptomatic enzyme elevation or radiologic abnormalities, while grade ≥ 3 indicates clinically significant disease. However, CTCAE does not incorporate comprehensive clinical and radiological diagnostic criteria and may fail to distinguish incidental enzyme elevations from true inflammatory injury—an important limitation given the frequent occurrence of asymptomatic enzyme elevations during ICI therapy [156,157]. Consequently, misclassification may lead to inappropriate decisions, including unnecessary interruption or discontinuation of immunotherapy.

Similarly, biliary complications (e.g., cholangitis, cholecystitis) lack dedicated classification systems and are assessed using CTCAE alongside clinical, laboratory and imaging findings [71,157].

Given the limitations of current grading systems, a multidisciplinary approach and more integrated classification models combining CTCAE with clinical and radiological criteria are needed for accurate assessment and optimal management (Table 7 and Table 8).

### 4.5. Therapy of IMP and Immune-Mediated Cholangitis and Cholecystitis

#### 4.5.1. Therapy of IMP

The National Comprehensive Cancer Network (NCCN) does not recommend discontinuing immunotherapy or any specific treatment for grade 1 IMP [170]. A cohort of 82 patients showed that the use of crystalloids within 48 h of a recorded increase in lipase levels is associated with a lower risk of long-term side effects [156]. Most authors agree that symptomatic IMP, corresponding to grades G2–G4, should be managed by discontinuing immunotherapy and following the standard protocol, which includes intravenous hydration and analgesics [4,5]. There are no clear recommendations from the European Society for Medical Oncology (ESMO) or the American Society of Clinical Oncology (ASCO) regarding the use of corticosteroids, whereas the NCCN clearly recommends their introduction for IMP grade 3 or 4 if there is no improvement with the standard of care [4,5,170]. A temporary interruption of immunotherapy followed by the introduction of prednisone or methylprednisolone (0.5–1.0 mg/kg/day) is recommended for G3, while permanent interruption of immunotherapy and higher doses of corticosteroid therapy (1–2 mg/kg/day) are recommended for G4 [142,170]. Hori Y et al. reported absolute success rates in treating patients with severe IMP using corticosteroids [142]. After clinical recovery to grade ≤ 1, gradual de-escalation over 4 to 6 weeks is recommended [4]. There is no universally accepted method of tapering. If symptoms worsen, the dose is increased to the previously effective level. To note, some studies showed that corticosteroid use did not affect the duration of the acute phase of IMP, biochemical or radiological parameters, prevent long-term side effects or influence survival [156,166].

Following corticosteroid therapy, IMP has been shown to be reversible in most cases (83.6%), although recurrence and mortality rates of 8.2% each have been reported [171]. A subset of patients fails to achieve a clinical response to corticosteroid therapy, referred to as steroid-refractory disease, while others experience symptom recurrence upon dose tapering, defined as steroid-dependent disease. Despite these challenges, the incidence of both forms remains relatively low among patients with IMP, indicating that most patients respond adequately to standard corticosteroid treatment. In cases where corticosteroids are contraindicated, or in steroid-refractory steroid-dependent disease, immunosuppressive agents are used as alternative therapy; however, recommendations for using immune-modulating agents are extrapolated from evidence for treating autoimmune diseases. The most commonly reported agents in the literature include MMF and biologic therapies such as IFX, rituximab and tacrolimus, which are used as adjunctive immunomodulatory treatments [162,163,172,173,174,175]. The use of IFX in the context of IMP therapy is considered safe; moreover, preclinical studies suggest that IFX may enhance the antitumor efficacy of ICIs [176]. According to NCCN guidelines in grade 4 IMP, if there is no clinical improvement within 48 h of starting corticosteroid therapy, treatment with IFX should be initiated. Following an initial dose of 5 mg/kg, a repeat dose may be administered after two weeks. This dosing strategy is not formally established in current guidelines but is based on case reports [177]. Currently, there is a lack of robust prospective studies to clearly define the optimal dosing, duration and treatment protocols for these agents in IMP, as available evidence is largely based on small case series and retrospective analyses. A proposed treatment algorithm for immune-mediated pancreatitis according to severity is shown in Table 9.

#### 4.5.2. Treatment of Immune-Mediated Cholangitis and Cholecystitis

Treatment of Immune-Mediated Cholangitis is based on discontinuation of immunotherapy and initiation of UDCA and immunosuppressive therapy [178]. As the first line of immunosuppressive therapy, prednisone is prescribed at a dose of 0.5–2 mg/kg/day, or budesonide at a dose of 3 × 3 mg/day. UDCA is most commonly administered alongside corticosteroids at doses ranging from 250 to 1500 mg daily. UDCA is recommended for its established cytoprotective effect and its stimulation of biliary secretion [145,146]. Following the discontinuation of corticosteroids, long-term continuation of UDCA therapy is recommended. Unlike corticosteroids, which are not advisable for prolonged use, UDCA can be safely administered over extended periods without significant adverse effects, while contributing to a further reduction in serum ALP levels [146]. Although the efficacy of corticosteroids in treating Immune-Mediated Cholangitis is low (11.5%), they remain the first-line therapy. Among immunosuppressive agents, azathioprine (50 mg), MMF (2 g), tacrolimus (2 mg) and tocilizumab (4 mg/kg) have been used [178,179,180].

The treatment of Immune-Mediated Cholecystitis follows standard cholecystitis treatment guidelines, while the decision to discontinue immunotherapy is made on an individual basis, depending on the severity of the clinical presentation and any complications that develop. As this is a rare occurrence, there are no clear recommendations regarding ICI cessation or the potential role of corticosteroids [150].

### 4.6. Long-Term Consequences of Immune-Mediated Pancreatobiliary Complications and Influence on Further Therapy

Although many patients initially present with mild or even subclinical pancreatobiliary complications associated with ICI therapy, it has become increasingly clear that some do not fully recover and may develop persistent or irreversible organ damage. In practice, this can significantly influence both quality of life and subsequent oncological therapy [156,178].

The most common long-term complications of IMP are exocrine pancreatic insufficiency in about 3% of cases and diabetes mellitus in around 9% of cases [181]. Exocrine insufficiency should be managed with early and continuous pancreatic enzyme replacement therapy, while diabetes, which frequently presents as diabetic ketoacidosis in up to half of cases, typically requires insulin treatment [182].

However, it appears that some long-term consequences can be prevented. A study conducted on a cohort of 82 patients with IMP showed that early administration of intravenous crystalloids within 48 h of lipase elevation is associated with a significantly reduced risk of long-term adverse outcomes, while 24% of patients who continued immunotherapy without intravenous hydration experienced persistent adverse effects [156].

Although the NCCN recommends initiating corticosteroids in grade 3–4 disease if there is no improvement with standard therapy, evidence on long-term benefit remains conflicting [163,165].

Limited evidence of corticosteroid effectiveness must be balanced with their adverse event profile, as long-term use of corticosteroids may worsen survival and promote tumor progression through metabolic, hormonal and microbiome-related effects [183]. Furthermore, long-term corticosteroid therapy is associated with a wide range of serious complications, including osteoporosis, diabetes mellitus, cardiovascular events, infections, psychiatric disorders and avascular necrosis, with risks increasing with higher cumulative doses [184].

While re-challenge with ICI is possible, at least one-quarter of patients (23.5%) who are re-challenged after an episode of hepatobiliary injury, including cholangitis, experience recurrence of liver or biliary tract toxicity [185].

## 5. Multidisciplinary Management

Management of irAEs is best coordinated by the treating oncology team in collaboration with hepatology or gastroenterology, with input from other relevant specialties as required [186,187,188]. Decisions regarding ICI interruption, continuation or rechallenge should take into account the severity of toxicity and balance the risk of recurrent organ injury against the anticipated oncological benefit [5,186]. Hepatologists and gastroenterologists contribute to the diagnostic work-up and guide immunosuppressive or supportive treatment according to the pattern and severity of organ injury [18,172,186,187].

Radiological imaging is primarily used to identify alternative or coexisting causes, including tumor progression, biliary obstruction, vascular disease and structural hepatobiliary or pancreatic abnormalities [5,172,186]. In patients with a predominantly cholestatic pattern, MRCP can demonstrate features associated with Immune-Mediated Cholangitis, including bile duct dilatation, stenosis, wall thickening and irregularity [186]. EUS may be considered when biliary obstruction remains a concern, whereas ERCP is generally reserved for patients in whom imaging demonstrates an anatomical obstruction or stricture that may require intervention [18]. Liver biopsy may provide additional diagnostic information when the diagnosis remains uncertain, or the presentation is atypical or refractory to initial treatment [18,186].

Pre-existing liver disease can complicate the evaluation of abnormal liver tests during ICI therapy, and hepatology consultation may be considered when such patients develop suspected IMH [172,186]. Early specialist consultation is particularly relevant in high-grade or steroid-refractory IMH [18,187]. Structured multidisciplinary models may also provide more timely access to specialist input and facilitate the diagnosis and management of complex immune-related toxicities [186,188].

**Figure 2:**
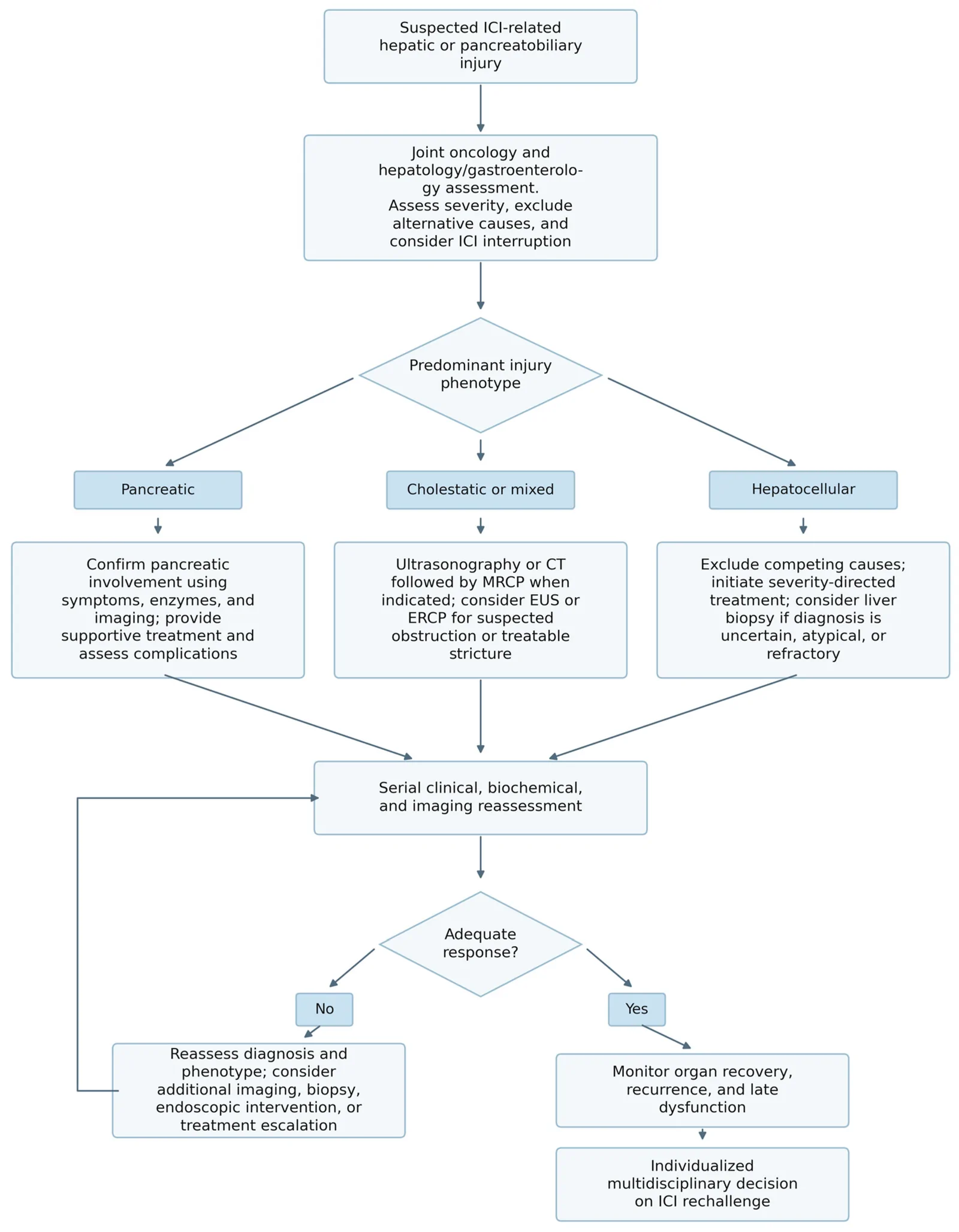
Proposed multidisciplinary algorithm for the evaluation and management of ICI-mediated hepatic and pancreatobiliary complications (ERCP—endoscopic retrograde cholangiopancreatography, MRCP—magnetic resonance cholangiopancreatography).

Collaboration should continue beyond the acute presentation to monitor clinical and biochemical recovery, identify recurrent or persistent injury and determine whether further immunosuppressive, endoscopic or other phenotype-directed management is required [18,172,186]. Following resolution of liver injury, any decision to rechallenge with an ICI should follow toxicity-specific guidance and be individualized through multidisciplinary assessment. A proposed multidisciplinary workflow integrating diagnostic evaluation, phenotype-directed management, response assessment and follow-up is presented in Figure 2.

## 6. Future Perspectives

Current research is increasingly investigating predictive biomarkers, including genetic, cytokine and immune profiling of patients and corresponding tumors, as well as microbiome analysis and circulating antibodies. Machine learning-based predictive models may play an important role in identifying patients at higher risk of developing immune-related adverse events, thereby enabling more personalized immunotherapy selection, earlier surveillance and potential prophylactic interventions aimed at reducing toxicity.

Further advances in understanding the pathophysiological mechanisms underlying ICI-induced toxicities may also facilitate the development of new targeted therapeutic strategies. Substantial variability still exists in clinical practice regarding diagnostic approaches, steroid tapering duration, immunosuppressive escalation and de-escalation strategies and decisions surrounding ICI rechallenge. Future large-scale prospective studies and international consensus guidelines will therefore be essential to standardize management and improve patient outcomes.

## 7. Conclusions

Improving awareness, strengthening multidisciplinary collaboration and developing standardized management pathways are essential to ensure timely intervention, maintain the benefits of immunotherapy and minimize treatment-related morbidity. A better understanding of the pathophysiological mechanisms of malignant diseases and ICI-mediated toxicities from clinical and basic studies, alongside the development of machine learning-based models represent the future of oncology management. Until then, further prospective studies are needed to better define optimal diagnostic and therapeutic strategies for all irAEs of ICI therapy to achieve a better balance between toxicity management and cancer control.

## Figures and Tables

**Table 1:** Incidence of IMH among individual ICIs [22,23,24,25,26,27,28,29,30,31,32,33,34,35,36,37,38,39,40].

Drug	ICI Class	All-Grade ICI-Mediated Hepatitis (%)	Grade ≥ 3 ICI-Mediated Hepatitis (%)	References	Reported Hepatitis-Related Adverse Events in the FAERS Database (*n*) *
Ipilimumab	CTLA-4 inhibitor	1–14	0.5–12	[20,21,22,23]	76
Nivolumab	PD-1 inhibitor	0.5–2.5	0.5–1	[24,25,26]	247
Cemiplimab	PD-1 inhibitor	0.5–1.5	0.5–1.5	[27,28,29]	19
Pembrolizumab	PD-1 inhibitor	1.5–9	1.5–2	[22,30,31,32]	268
Avelumab	PD-L1 inhibitor	5.5	4	[33]	12
Durvalumab	PD-L1 inhibitor	0.3	0.3	[34]	18
Nivolumab + Ipilimumab	Combination	2–20	0.3–20	[25,35,36]	399
Pembrolizumab + Ipilimumab	Combination	2–10	1.5–6	[37,38]	17

* Data was extracted from the pharmacovigilance study by Fu et al. [41].

**Table 2:** Predictive biomarkers and their significance in recent studies (↑: elevated).

Biomarker Category	Specific Factor/Biomarker	Evidence Level	Significance	Reference
General factors	Prior ICI therapy	Better-supported	OR = 3.58 (95% CI: 2.08–6.14)	[21]
	Age 56–63 years	Better-supported	MD = −5.09 (95% CI: −9.52 to −0.67)	[21]
	Female sex	Better-supported	Increased risk, *p* = 0.038	[21]
Genetic factors	HLA-DR4	Emerging/ Exploratory	53% prevalence	[64]
	EDIL3, SEMA5A variants	Emerging/ Exploratory	OR = 2.08–2.4 (*p* = 0.01)	[69]
	GABRP, SMAD3, SLCO1B1	Emerging/ Exploratory	OR = 2.08–2.4 (*p* = 0.01)	[69]
	Traditional AIH HLA alleles	Inconclusive	Not overrepresented	[63]
Underlying liver disease	NAFLD/MAFLD	Emerging/ Exploratory	Significant in Cox hazard analysis (HR not reported)	[52]
	AIH, PBC, PSC (PD-1/PD-L1)	Better-supported	No increased risk (0/22 grade ≥ 3 irAEs)	[21]
	Alcohol consumption	Better-supported	Associated with steroid unresponsiveness	[53]
Immunological factors	CD8+ T cells (↑ perforin, granzyme B, ICOS, HLA-DR)	Emerging/ Exploratory	Significantly elevated (*p* = 0.05)	[61]
	Circulating CD8+ EM (CD38 + HLA-DR + CXCR3+)	Promising/Emerging	Distinguishes ICI hepatitis from DILI/AIH	[62]
	CCR2+/CD163+ monocytes	Emerging/ Exploratory	Significantly elevated (*p* = 0.0001)	[48]
	Pre-existing ANA positivity	Inconclusive	Inconclusive association	[60,63]
	Composite cytokine score (CYTOX)	Emerging/ Exploratory	AUC = 0.68–0.70	[65]
	↑ IL-6, IL-17f (Th17); IL-5, IL-13 (Th2)	Emerging/ Exploratory	Th17/Th2 skewing (significant)	[59]

**Table 3:** Recommended diagnostic workup in patients with acute hepatitis during ICI therapy.

	Diagnostic Workup
**Imaging**	Abdominal ultrasound with Doppler or/and CT or MRCP to exclude: • heterogeneous liver parenchyma • liver metastases • portal vein and/or hepatic vein thrombosis • bile duct dilatation or irregularities
**Virology**	Serology: HAV, HBV, HCV, HEV, CMV, EBV, HSV-1 and HSV-2
**Thyroid hormones**	TSH, T3, T4
**Immunology**	Autoantibodies: • antinuclear antibodies (ANAs) • smooth muscle/anti-actin antibodies (SMAs) • antimitochondrial antibodies (AMAs) • liver-kidney microsomal type 1 antibodies (anti-LKM-1) • soluble liver antigen/liver-pancreas antibodies (anti-SLA/LP) • liver cytosol type 1 antibodies (anti-LC1) Immunoglobulins: IgG, IgA, IgM
**Bacterial infections**	Urine and blood cultures Chest X-ray
**Toxic causes**	Detailed medication history (current and prior) Dietary supplements Assessment of alcohol intake Urine toxicology screen
**Other causes**	Ischemic liver disease Congestive hepatopathy Hemochromatosis Wilson disease

**Table 4:** Grading of ICI-related hepatotoxicity according to National Cancer Institute Common Terminology Criteria for Adverse Events [71] (ULN—upper limit of normal, ADL—activities of daily living, DILI—drug-induced liver disease).

Parameter	Grade 1	Grade 2	Grade 3	Grade 4	Grade 5
**ALP**	>ULN to 2.5× ULN; 2 to 2.5× baseline if abnormal	>2.5 to 5× ULN; >2.5 to 5× baseline if abnormal	>5 to 20× ULN; >5 to 20× baseline if abnormal	>20× ULN; >20× baseline if abnormal	-
**ALT**	>ULN to 3× ULN; 1.5 to 3× baseline if abnormal	>3 to 5× ULN; >3 to 5× baseline if abnormal	>5 to 20× ULN; >5 to 20× baseline if abnormal	>20× ULN; >20× baseline if abnormal	-
**AST**	>ULN to 3× ULN; 1.5 to 3× baseline if abnormal	>3 to 5× ULN; >3 to 5× baseline if abnormal	>5 to 20× ULN; >5 to 20× baseline if abnormal	>20× ULN; >20× baseline if abnormal	-
**Bilirubin**	>ULN to 1.5× ULN; >1 to 1.5× baseline if abnormal	>1.5 to 3× ULN; >1.5 to 3× baseline if abnormal	>3 to 10× ULN; >3 to 10× baseline if abnormal	>10× ULN; >10× baseline if abnormal	-
**GGT**	>ULN to 2.5× ULN; 2 to 2.5× baseline if abnormal	>2.5 to 5× ULN; >2.5 to 5× baseline if abnormal	>5 to 20× ULN; >5 to 20× baseline if abnormal	>20× ULN; >20× baseline if abnormal	-
**Liver failure**	-	-	Asterixis, mild encephalopathy, DILI, coagulopathy, limitation in self-care ADL	Life-threatening consequences; severe encephalopathy; coma	Death
**Portal hypertension**	-	Reduced portal venous flow	Reversed portal venous flow with varices and/or ascites, more severe hepatic encephalopathy	Life-threatening consequences; urgent intervention indicated	Death

**Table 5:** Management of IMH according to severity [18] (LFTs—liver function tests, MMF—mycophenolate mofetil).

**Grade I**	⮚ ICI: Continue; consider holding if concerning upward trend ⮚ Corticosteroids: None—observe ⮚ Monitoring: liver function tests (LFTs) 1–2×/week ⮚ If stable → continue ICI with monitoring ⮚ If worsening → regrade and escalate
**Grade II**	⮚ ICI: Hold ⮚ Corticosteroids: Prednisone 0.5–1 mg/kg/day if symptomatic or no improvement in 1–2 weeks ⮚ Monitoring: LFTs every 3–5 days; check INR periodically ⮚ If improves to G1 → taper corticosteroids over ≥ 4 weeks → resume ICI when ALT/AST ≤ baseline and prednisone ≤ 10
**Grade III**	⮚ ICI: Hold ⮚ Corticosteroids: Prednisone/methylprednisolone 0.5–1 mg/kg/day (max 60 mg) ⮚ Monitoring: LFTs every 1–5 days; consider liver biopsy ⮚ Evaluate response at 48–72 h: ⮚ Responding (≥50% drop in transaminases): ⮚ Continue corticosteroids → taper over 6–8 weeks once sustained G1 ⮚ Consider ICI rechallenge with anti-PD-1 monotherapy (recurrence ~35%) ⮚ Not responding (steroid-refractory): Add MMF OR tacrolimus (lowest effective dose) ⮚ If still refractory → tocilizumab or ATG ⮚ Escalate to G4 protocol if worsening
**Grade IV**	⮚ ICI: Permanently discontinue ⮚ Corticosteroids: Methylprednisolone 1–2 mg/kg/day IV; consider early concomitant MMF ⮚ Monitoring: LFTs every 1–3 days; hospitalize ⮚ Evaluate response at 72 h ⮚ Responding (≥50% drop in transaminases): Continue IV corticosteroids → convert to oral → taper over 8+ weeks ⮚ Not responding: Add MMF or tacrolimus ⮚ If refractory → tocilizumab ⮚ Fulminant cases → ATG; evaluate for liver transplant Do NOT rechallenge ICI if synthetic dysfunction (bilirubin > 2.5 mg/dL + INR > 1.5, ascites or encephalopathy)

**Table 6:** Clinical and diagnostic aspects in the diagnosis of IMP.

Diagnostic Criteria	Revised Atlanta Classification for IMP (Two Out of Three Criteria) [138]
**Clinical**	Typical abdominal pain—starting from epigastrium and radiating to the back
2. **Biochemical**	Minimum of 3× increase in serum pancreatic enzymes (lipase—the more accurate marker)
3. **Imaging**	Abdominal MSCT [132,139,140] milder interstitial form (pancreatic enlargement, segmental reduction in contrast enhancement of the pancreatic parenchyma, peripancreatic fat stranding or pancreatic enlargement with heterogeneous contrast enhancement) acute necrotic or acute peripancreatic collection (rare form) Abdominal MRI [135] reduced signal intensity and restricted diffusion late-phase enhancement with gadolinium, diffusion restriction on diffusion-weighted imaging, main pancreatic duct narrowing in head (differential diagnosis AIP) EUS [133] diffuse hypoechoic pancreas enlargement patchy and heterogeneous parenchymal pattern PET-CT [143] high 18F-fluorodeoxyglucose (FDG) avidity diffuse uptake typical focal presentations—diagnostic challenge for malignancy, pancreatic metastases

**Table 7:** CTCAE classification of IMP severity.

Grade	Description
**Grade 1 (G1)**	–
**Grade 2 (G2)**	Enzyme elevation; radiologic findings only
**Grade 3 (G3)**	Severe pain; vomiting; medical intervention indicated (e.g., analgesia, nutritional support)
**Grade 4 (G4)**	Life-threatening consequences; urgent intervention indicated
**Grade 5 (G5)**	Death

**Table 8:** CTCAE classification of Immune-Mediated Cholangitis and Cholecystitis severity.

Grade	Description
**Grade 1 (G1)**	–
**Grade 2 (G2)**	Symptomatic; medical intervention indicated
**Grade 3 (G3)**	Severe symptoms; invasive intervention indicated
**Grade 4 (G4)**	Life-threatening consequences; urgent operative intervention indicated
**Grade 5 (G5)**	Death

**Table 9:** Treatment of IMP according to severity [4,5,170].

**Grade I**	⮚ ICI: Continue; consider holding if concerning upward trend ⮚ Corticosteroids: None—observe ⮚ Best supportive care ⮚ If stable → continue ICI with monitoring ⮚ If worsening → regrade and escalate
**Grade II**	⮚ ICI: Hold ⮚ Corticosteroids: none—observe ⮚ Intravenous crystalloids and analgetics (best supportive care) ⮚ If improves to G1 → resume ICI
**Grade III**	⮚ ICI: Hold ⮚ Corticosteroids: Prednisone/methylprednisolone 0.5–1 mg/kg/day ⮚ Intravenous crystalloids and analgetics (best supportive care) ⮚ Monitoring—if recovery to grade ≤ 1, gradual de-escalation of corticosteroids over 4 to 6 weeks is recommended ⮚ Escalate to G4 protocol if worsening
**Grade IV**	⮚ ICI: Permanently discontinue ⮚ Corticosteroids: Methylprednisolone 1–2 mg/kg/day IV ⮚ Monitoring—if recovery to grade ≤ 1, gradual de-escalation of corticosteroids over 4 to 6 weeks is recommended ⮚ While taper symptoms worsen—corticosteroid dose is increased to the previously effective level ⮚ Not responding within 48 h add IFX (5 mg/kg every 2 weeks) ⮚ Alternative therapies—MMF/tacrolimus/rituximab

## Data Availability

No new data were created or analyzed in this study. Data sharing is not applicable to this article.

## Abbreviations

The following abbreviations are used in this manuscript:

AIH	Autoimmune hepatitis
AIP	Autoimmune pancreatitis
ANA	Antinuclear antibodies
AMA	Antimitochondrial antibodies
ALT	Alanine aminotransferase
AST	Aspartate aminotransferase
ATG	Antithymocyte globulin
CRP	C-reactive protein
CT	Computed tomography
CTCAE	Common Terminology for Clinical Adverse Events
CTLA-4	Cytotoxic T-lymphocyte-associated antigen 4
DAA	Direct-acting antivirals
DILI	Drug induced liver disease
ERCP	Endoscopic retrograde cholangiopancreatography
EUS	Endoscopic ultrasound
EUS-FNA	EUS fine-needle aspiration
EUS-FNB	EUS fine-needle biopsy
HCC	Hepatocellular carcinoma
HBV	Hepatitis B virus
HCV	Hepatitis C virus
HIV	Human immunodeficiency virus
ICI	Immune checkpoint inhibitor
IFX	Infliximab
IgG	Immunoglobulin G
IgG4-SC	IgG4-related sclerosing cholangitis
IL-2	Interleukin-2
IMH	Immune-Mediated Hepatitis
IMP	Immune-Mediated Pancreatitis
INR	International Normalized Ratio
irAEs	Immune-related adverse events
LFT	Liver function tests
LKM-1	Liver-kidney microsomal type 1 antibodies
LT	Liver transplantation
MASLD	Metabolic-associated steatotic liver disease
MMF	Mycophenolate mofetil
MRCP	Magnetic resonance cholangiopancreatography
MRI	Magnetic resonance imaging
MSI	Microsatellite instability
NSCLC	Non-small cell lung cancer
PBC	Primary biliary cholangitis
PD-1	Programmed cell death protein 1
PD-L1	Programmed death ligand 1
PET-CT	Positron emission tomography-computed tomography
PLWH	People living with HIV
PSC	Primary sclerosing cholangitis
RCC	Renal cell carcinoma
SMA	Smooth muscle/anti-actin antibodies
SNPs	Single-nucleotide polymorphisms
TLRs	Toll-like receptors
TNF-α	Tumor necrosis factor α
UDCA	Ursodeoxycholic acid
